# Therapeutical Application

**Published:** 1862-03

**Authors:** 


Iodine.—The following cn the *Therapeutic application* of iodine is a continuation of the selection in the last number of the Register, from Dr. Percy's lecture:

*Therapeutical Application.*—Although iodine was discovered by Courtois in 1812, it was not used in medicine until 1820; on 25th July in that year Dr. Coindet, of Geneva, read a paper before the Society of Natural Sciences of Geneva on the use of iodine in the cure of goitre. He was led to investigate the action of iodine on goitre from the known beneficial effects of burnt sponge in that disease, the curative effects of which were entirely owing to the small amounts of iodides and iodates contained in the ashes. As iodine was found so efficacious in goitre, it was soon used with equally beneficial results in scrofula; and to Brera, Lugol and Manson, we owe much of the knowledge we now possess of it in this disease. As it was a new remedy, and really possessed extraordinary and valuable powers, it was employed by many in every kind of disease, and by some vaunted as a universal specific, and by others condemned as injurious and useless; but as we learn more of its physiological action and modus operandi, we know better how to determine its real value. Dr. Williams, of the London College of Pharmacy, first announced its great value in the treatment of the tertiary form of syphilis in 1834.

*Local Effects.*—Iodine is generally used locally, either in the form of tincture, compound tincure, or in solution in glycerine or collodion; we will give you the most appropriate formulæ for preparing these solutions in the proper place. Iodine was first used as an external application in goitre, and several cases were cured by this means without its internal administration. It has been for many years extensively used as a local application to glandular enlargements, especially those in the various forms of scrofulous disease. It is a very common thing to see children of a scrofulous diathesis with enlarged lymphatic glands, and those about the neck are more frequently diseased than in other parts of the body. Although from experience the physician knows that the local application of iodine is of great service in the treatment of these enlargements, he is frequently prevented from applying it because it leaves a yellow, unpleasant-looking stain upon the skin. This appears in some instances to be an objection to its use, for young ladies are unwilling to have so conspicuous a mark upon them, but this difficulty may be nearly always overcome by wearing a broad velvet band around the neck, and upon the spot where the band covers the tumor a piece of oiled silk should be placed; this cover of oiled silk assists the action of the iodine.

It has been used very extensively of late years as a local application in strumous ophthalmia. In this disease the little patients are very frequently troubled with great intolerance of light; in addition to the other treatment that is required, tincture of iodine is applied over the orbit and occasionally around the eye, and very great benefit is experienced from the local application. I have on several occasions seen perfect relief within twenty-four hours, by the local application of iodine alone in this photophobia scrofulosa. I have no doubt you all avail yourselves of the excellent opportunities afforded you for instruction at the New York Eye and Ear Infirmary; you also have peculiar and unusual opportunities of studying diseases of the eye and ear under the able teachings and clinical explanations of your earnest and learned Professor of Ophthalmic and Aural Surgery. You have at these clinics, and at those of the Eye Infirmary, seen many little patients whose first appearance denoted the trouble under which they were laboring. Every effort is made by them to exclude the light; and the hanging head, knit brow, and elevated upper lip and nose, are legible marks of this strumous ophthalmia, accompanied with photophobia. In many of these cases you will be astonished to see such an amount of intolerance to light, with so little visible symptoms of disease within the eye itself. In these cases you will find marked benefit by the local application of compound tincture of iodine over the orbit and around the eye; underneath the eye you should make but one slight and quick application of it, but over the orbit apply it until the skin is deeply colored with it, and over the whole make one application of iodine in collodion. Insist upon the child being kept as much as possible out of doors, and you will frequently see in twenty-four hours a removal of the unpleasant symptoms. In scrofulous otorrhœa local application of the same substances behind the ear are equally beneficial as in diseases of the eye, but in both of these affections be careful not to apply the iodine on the inflamed and excoriated skin over which the unhealthy discharge has been flowing; and above all, be careful not to let the tincture run into the eye. In scrofulous persons the tonsils are nearly always enlarged; a local, internal application of tincture of iodine is generally more successful in removing the enlargement than any other application. But there are many instances, with children, where an internal application can not be made; in these cases an external application over the tonsils will in time relieve the difficulty. In swellings about the large joints, especially those of a chronic character, free and frequent application of the iodine will be found of great advantage. It will be equally serviceable also in the swelling of the smaller joints and in paronychiæ. It is frequently used with advantage to swollen bursæ, corns, chilblains, furuncles, etc. When thoroughly applied in the first stage of non-syphilitic inflammation of the inguinal glands, it will generally check the inflammation and prevent suppuration. In the early stage of inflammation of the breast it will frequently arrest its progress. It has been recommended on good authority as an excellent application directly to the wound in the bites of snakes and venomous reptiles.

In the treatment with external applications of iodine, the success will depend upon the method in which it is applied, as well as in the preparation made use of. For ordinary application to adults, both the tincture and the compound tincture are too weak in iodine, and I am in the habit of preparing a compound tincture in preference to a simple tincture, as the iodide of potassium makes the iodine more soluble not only in the alcohol, but by the absorbents of the skin. The officinal formula directs that half an ounce of iodine, and one ounce of iodide of potassium, be dissolved in one pint of alcohol; but for external use, this contains an unnecessarily large proportion of iodide of potassium, and much too small a quantity of iodine. The preparation I usually use is made by dissolving half an ounce of iodide of potassium, and an ounce and a half of iodine, in a pint of alcohol of about eighty-six per cent. If but a mild effect is wished, one application of this with a camel's hair pencil over the surface will be sufficient; but in many instances it will be necessary to renew it two or three times, merely waiting until the previous application is dry, and this may be repeated at first twice in twenty-four hours, afterwards, once a day or once in two days as required, but often enough to produce free and frequent exfoliation of the skin. If but one application of the iodine is needed, or if the application is made at long intervals, I usually apply over the spot painted by the tincture a good coating of iodinal collodion, made after the following formula:—Take of iodine, two drachms: Canada balsam, one drachm; collodion, four oz.; dissolve first the iodine, then the Canada balsam in the collodion. The cork of the bottle should be perforated by a peg of wood, to which a camel's hair brush should be attached. Some persons use this collodion without any previous application of the tincture, but I think a better effect is produced by applying the tincture first, and then coating it with collodion. By this means the pores of the skin are not immediately closed, and the iodine exerts a more energetic effect, and the evaporation of the iodine is to some extent prevented by the outer covering of collodion. The balsam is added to prevent its cracking. But if a daily application of iodine is needed, it is not well to paint it over with the iodinal collodion, because so long as the collodion remains, it will prevent the action of the next application of iodine on the skin. In cases of synovitis tonsilitis, etc., it is better to use the compound tincture I have mentioned, and then cover the spot with a piece of oiled silk, and cover the silk with a bat of cotton. I am satisfied I have discussed many a non-syphilitic bubo by this means, and quickly relieved cases of synovitis. In the photophobia scrofulosa of which I have spoken, I generally, if possible, apply my compound tincture over the brow until it is of a dark color; two successive applications will generally be sufficient; but sometimes I apply a third, waiting a minute or two for the second to dry; over this spot I then apply a piece of oiled silk, and over this a bat of cotton, the whole being kept in place by two or three strips of isinglass plaster. There will be a burning pain for a short time, but it soon subsides, and this pain may be to a great extent prevented by smearing the spot slightly with castor oil before applying t e oiled silk. The same plan may be adopted over the tonsils, behind the ear, or any spot where it is difficult to keep the oiled silk on.

Of late years, glycerine has been added to the compound tincture of iodine, to prevent its drying so quickly, when used as an external application, and in some instances is very beneficial, especially where it is desired to cover the part with oiled silk. From one to two drachms of glycerine may be added to an ounce of the compound tincture. It is more serviceable when applied the last time, just before covering it with the silk.—*American Medical Times.*

